# Clinical features and novel pathogenic variants of patients with Behçet’s disease like trisomy 8

**DOI:** 10.1186/s13023-025-03878-y

**Published:** 2025-07-04

**Authors:** Xingru Ding, Jinghan Yang, Xiao Han, Yi-Fan Shen, Kangkang Yang, Yaoyao Shangguan, Yiwei Dong, Xiaohua Ye

**Affiliations:** 1 Department of Pediatric Rheumatology, The Second Affiliated Hospital, Yuying Children’s Hospital of Wenzhou Medical University, Wenzhou, Zhejiang China; 2 School of Medicine, The Chinese University of Hong Kong, Shenzhen, 518172 Guangdong China; 3 Zhejiang Provincial Clinical Research Center for Pediatric Precision Medicine, The Second Affiliated Hospital and Yuying Children’s Hospital of Wenzhou Medical University, Wenzhou, Zhejiang China

**Keywords:** Trisomy 8, Behçet’s disease, *NRAS*, Genetic sequencing, Chromosome abnormality

## Abstract

**Purpose:**

Chromosomal abnormalities, such as Trisomy 8 (T8), and genetic mutations may contribute to the unique clinical phenotype of Behçet’s Disease (BD). This study aims to characterize the clinical and genetic features of patients presenting with BD-like symptoms associated with T8 (T8-BD).

**Methods:**

We analyzed a cohort of 8 patients with T8-BD and associated genetic variants, including 1 newly identified case from our center and 7 previously reported in the literature. Genetic sequencing and karyotyping analyses were conducted, with Sanger sequencing used to confirm variants. We assessed clinical phenotypes, genetic backgrounds, treatments, and clinical outcomes.

**Results:**

This study comprised predominantly female (87.5%) and East Asian (87.5%) patients, spanning pediatric to elderly populations, with a high comorbidity prevalence (87.5%), including autoimmune lymphoproliferative syndrome (ALPS), myelodysplastic syndrome (MDS), atypical familial Mediterranean fever (FMF), primary myelofibrosis (PM), polycythemia vera (PV), and Townes-Brocks syndrome (TBS). Uniform presentations included oral aphthosis (100%), intestinal lesions (100%), with most cases exhibiting fever (85.7%), anemia (85.7%), and elevated C-reactive protein (CRP) levels (85.7%). Genetic variants were identified in *NRAS*, *JAK2*, *MEFV*, *PTPN11*, and *SALL1*, comprising 5 missense variants and 1 nonsense mutation, including a *de novo NRAS* mutation newly reported in a pediatric patient. Treatment involved glucocorticoids (GC) combined with immunosuppressants (33.3%), a combination of GC, immunosuppressants, and biologics (50%), and anti-oral aphthosis medications (16.7%), with most patients achieving remission, except for one fatal outcome.

**Conclusions:**

Patients with T8-BD and genetic mutations exhibit distinct clinical features. Greater clinical awareness of autoinflammatory syndromes, combined with genetic and chromosomal analysis, is recommended in patients with BD-like symptoms who do not fully meet BD diagnostic criteria, especially those presenting with oral ulcers and systemic inflammation. This approach may enhance diagnostic precision and inform tailored treatment strategies.

**Supplementary Information:**

The online version contains supplementary material available at 10.1186/s13023-025-03878-y.

## Introduction

Behçet’s Disease (BD) is a rare disorder that causes recurrent oral aphthous and genital ulcers, uveitis, and skin lesions [1]. It predominately affects young adults and is most prevalent in regions along the ancient “Silk Road” [2], particularly in Mediterranean, Middle East, and Far East [2]. The etiology remains obscure; however, there is a consensus regarding the interplay between environmental risk factors and genetic susceptibility [3]. BD was first comprehensively described by Benediktos Adamantiades and Hulusi Behçet in 1931 and 1937, highlighting a triad of oral aphthous ulcers, genital ulcers, and hypopyon [4, 5]. Since then, additional clinical features have been systematically reported, including ocular [6], articular [7], vascular [8], gastrointestinal [9], and neurological involvement [10]. BD is being increasingly recognized to share clinical manifestations with systematic autoinflammatory disease and vasculitis [11], which can affect multiple organs and contribute to various atypical symptoms [12], therefore increasing the heterogeneity of BD and making it more difficult to diagnose clinically [13].

BD is considered part of the polygenic autoinflammatory disease spectrum, as it overlaps with several monogenic autoinflammatory conditions in terms of clinical presentations and immune dysregulation [14–16]. Among the genetic risk factors, *HLA-B51* has been identified as the strongest genetic association with BD. However, it accounts for less than 20% of the total genetic susceptibility, suggesting that additional genetic contributors remain undiscovered [17, 18]. Two large genome-wide association studies (GWASs) of BD conducted in Turkey [19] and Japan [20] identified *interleukin (IL)-10* and *IL-23R/IL-12RB2* as non-HLA susceptibility loci for BD. Furthermore, pathway-based analyses of these GWAS datasets highlighted focal adhesion, mitogen-activated protein kinases (MAPK) signaling, and transforming growth factor beta (TGF-β) signaling pathways as key molecular pathways involved in BD pathogenesis [21].

Trisomy 8 (T8) is a rare chromosome number abnormality and can activate the abnormal inflammatory process and immune gene expression [22]. T8 is frequently found in hematological diseases like myelodysplasia syndrome (MDS) and acute myeloid leukemia (AML) [23], and the correlations between MDS with T8 (T8-MDS) and autoinflammatory condition, have been strongly recognized in the past few decades, BD especially [24]. The activation of the NF-κB pathway might be the underlying pathology [25]. Indeed, there might be a potential link between T8 and BD. In 1999, Japan researcher Nawata first reported a case of T8 with BD phenotypes, namely oral aphthous ulcers, genital ulcers, rash, and recurrent fever, and believed that T8 was associated with BD [26]. Thereafter, there have been studies reporting that autoinflammatory phenotypes resembling BD are associated with T8, and BD patients with T8 often present with fever, gastrointestinal involvement, and erythema nodosum [24, 27]. Given that the clinical spectrum of autoinflammatory phenotypes associated with T8 is not restricted to BD, a term named TRIAD (trisomy 8-associated autoinflammatory disease) has recently been proposed [28, 29]. The underlying mechanism of T8 in the pathogenesis of BD-like symptoms may extend beyond the simple overexpression of chromosome 8 genes, involving epigenetic alterations and dysregulated inflammatory pathways that contribute to multisystemic organ damage and impaired immune homeostasis [22, 30].

Numerous studies have focused on BD phenotypes solely in the patients of T8-MDS. However, the conditions of abnormal karyotypes without MDS are largely ignored. Furthermore, there have been no cohort studies investigating BD patients with T8 combined with genetic mutations. In this study, we summarized the clinical and genetic findings of eight patients of Behçet’ s disease like trisomy 8 (T8-BD) accompanied by genetic variants, aiming to increase clinician awareness of these conditions and improve early diagnosis of BD.

## Methods

### Subjects and study design

Patients diagnosed as T8-BD plus gene mutation at The Second Affiliated Hospital and Yuying Children’s Hospital of Wenzhou Medical University were enrolled in this study. The inclusion criteria was as followings: (1) BD diagnosis: satisfy at least one of five classification criteria, that is, Japan revised criteria [31], Chen and Zhang criteria (China) [32], International Criteria for BD (ICBD; if above 18 years of age) [33], Consensus classification criteria for pediatric BD (if under 18 years of age) [34], and revised International Study Group criteria (ISG) [35]; (2) T8 diagnosis: detection of gene/chromosomal aberrations by peripheral blood karyotyping, bone marrow karyotyping, or sequencing (high-throughput sequencing); (3) Mutation seizures: detection of variants by sequencing (high-throughput sequencing). Standardized case report form was utilized to record demographic information, genetic background, and clinical manifestations including symptoms, laboratory tests, treatments, and clinical outcomes.

The study strictly adhered to the Declaration of Helsinki and has been approved by the Institutional Review Board and the Medical Ethics Committee of The Second Affiliated Hospital and Yuying Children’s Hospital of Wenzhou Medical University (2021-K-327-02). Informed consent was obtained from the parents or patients (if more than 18 years old) for participation in this study.

### Genetic sequencing and bioinformatics

Genomic DNA was extracted from the EDTA-treated peripheral blood collected from the affected individual. Quality control was performed by Qubit 2.0 fluorimeter. Protein-coding exome enrichment was conducted using xGen Exome Research Panel v2.0 (IDT, Iowa, USA) to construct the whole exome library. High-throughput sequencing was performed by MGI NBSEQ-T7 sequencer, and covered more than 99% of target sequence. Single nucleotide polymorphisms (SNPs) and Indels were screened that high quality and reliable variants obtained. Variants with a minor allele frequency (MAF) less than 1% in 1000 Genomes (ftp://1000genomes.ebi.ac.uk/vol1/ftp), dbSNP (http://www.ncbi.nlm.nih.gov/snp), Exome Aggregation Consortium (ExAC, http://exac.broadinstitute.org/), and Genome Aggregation Database (gnomAD, https://gnomad.broadinstitute.org/) were filtered out. To assess the pathogenicity of candidate variants, SIFT (http://sift.jcvi.org/), Polyphen-2 (http://genetics.bwh.harvard.edu/pph2/), MutationTaster (http://mutationtaster.org/), and CADD (https://cadd.gs.washington.edu/) were employed.

The pathogenicity of each variant was further classified according to the American College of Medical Genetics and Genomics (ACMG) guideline [36], with reference to the Online Mendelian Inheritance in Man (OMIM, https://www.omim.org/), Human Phenotype Ontology (HPO, https://hpo.jax.org/app/), and ClinVar (http://www.ncbi.nlm.nih.gov/clinvar). Variants classified as “pathogenic” or “likely pathogenic” were considered disease-causing and subjected to further validation. Sanger sequencing was performed to confirm the high-throughput sequencing results. Additionally, multiple sequence alignment analysis was further conducted on NRAS G12A mutation.

### Literature review

**Fig. 1:**
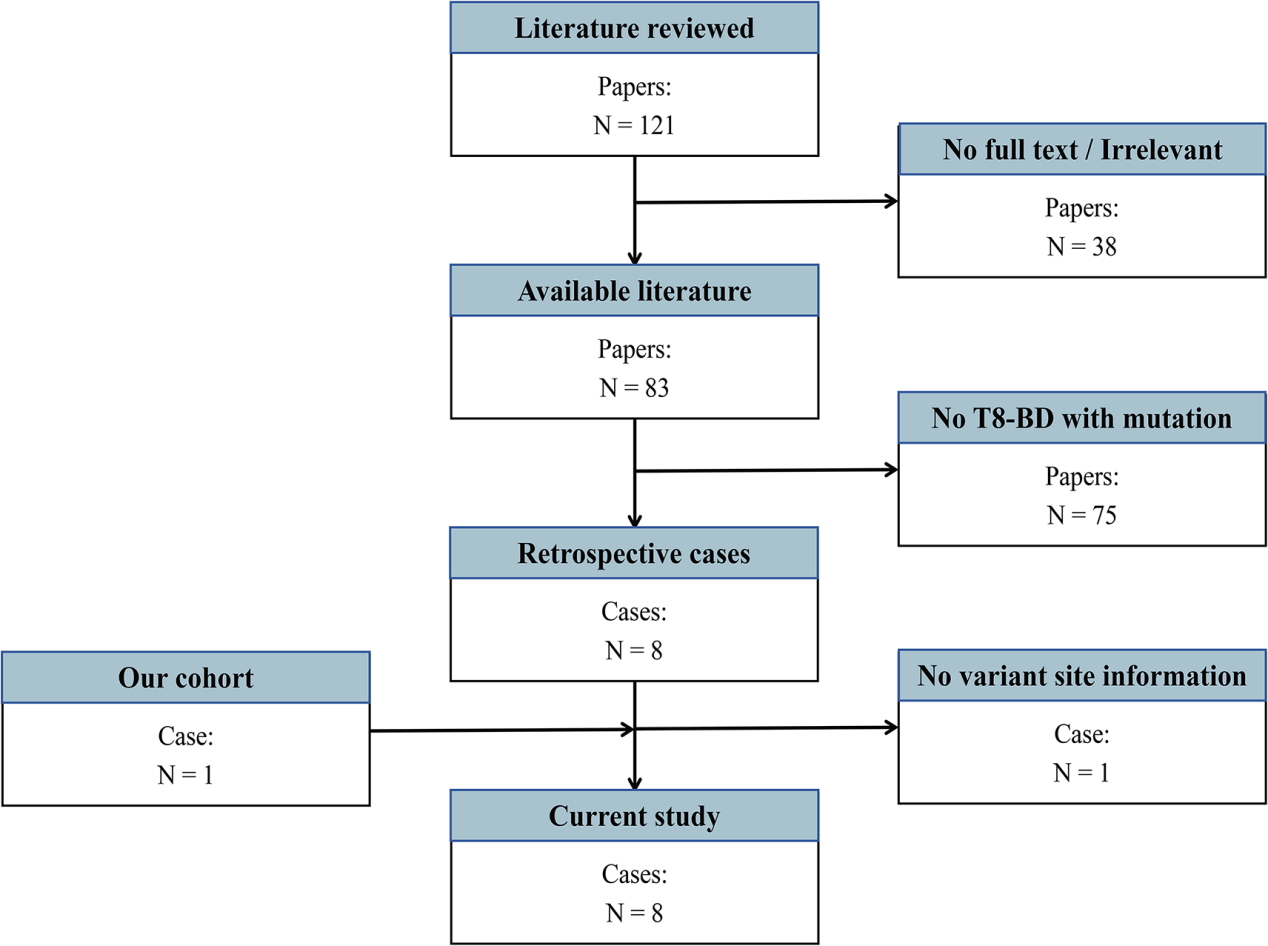
Flowchart of cohort participants inclusion in literature review

A systematic literature search including T8-BD patients accompanied by genetic mutations was performed. We searched PubMed, Scopus, web of science with the keyword combination (Behçet’s disease OR Behçet’s syndrome) AND trisomy 8 up to July 2024 without any restrictions. Reports with incomplete clinical data were excluded. Those simultaneously diagnosed with T8 and BD, and additionally accompanied with genetic variants were further critically filtered out by careful reading. We collected data including nation, gender, age at the first visit, age at the diagnosis of BD and T8, age at variant identification, comorbidities, clinical features, laboratory tests, medical regimen, and treatment outcome. The cohort enrollment flowchart is presented in Fig. 1.

### Statistical analysis

Statistical analyses were performed using SPSS version 27.0 (SPSS Inc., Chicago, IL, USA). Continuous variables, including age at the first visit, and age at diagnosis, were described as median and interquartile range (IQR). Categorical variables were summarized by percentages and frequencies. The data with missing information were excluded from calculation when describing the results.

## Results

### Clinical findings

**Fig. 2:**
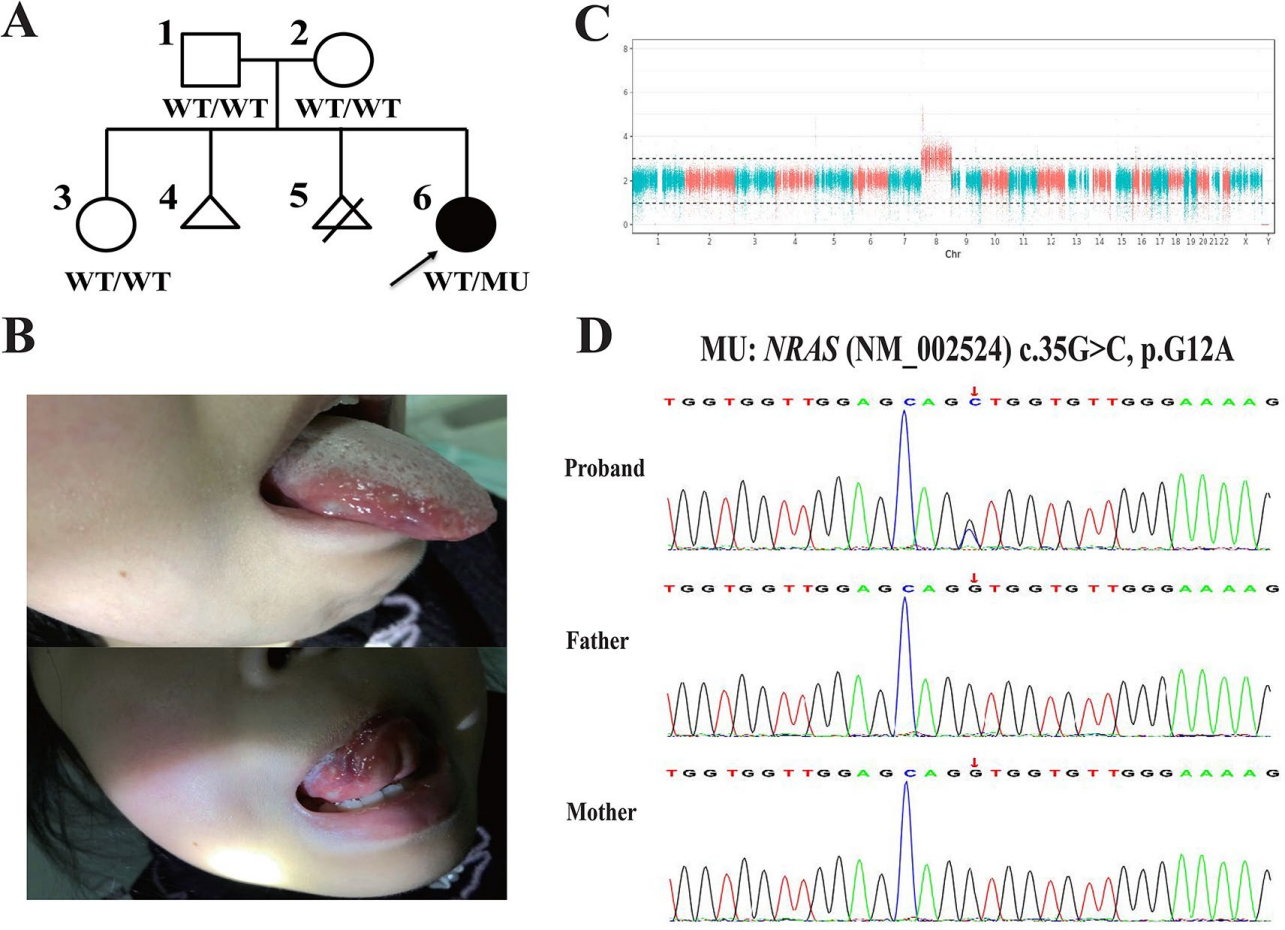
Clinical and genetic features of the reported case (P1) in this study. **(A)** Pedigree of the affected family. Males and females were represented by squares and circles, respectively. The triangle represents spontaneous abortion, and the triangle with a slash indicates terminated abortion. The filled symbol denoted affected patients, and the arrow indicated the proband. WT, wild-type; MU, mutant. **(B)** Oral aphthosis identified in P1. Multiple ulcers can be found on the lateral borders and the underside of the tongue. **(C)** Results of chromosome abnormality detection in P1. Chromosome number map showed the haploid duplication of chromosome 8. **(D)** DNA sequencing profiles of P1 and her parents. The parents’ normal sequences are shown in the upper panel, while the DNA sequence of P1 is shown in the lower panel

In total, 8 patients, including 7 patients reviewed from literature and 1 patient accompanied by one *de novo NRAS* mutation at The Second Affiliated Hospital and Yuying Children’s Hospital of Wenzhou Medical University newly reported this time, were enrolled. Out of them, there were 7 female probands (87.5%) and 1 male proband (12.5%), with a majority form East Asia (87.5%) and combining with comorbidities (87.5%), including 1 case of autoimmune lymphoproliferative syndrome (ALPS) [37], 2 myelodysplastic syndrome (MDS) plus atypical familial mediterranean fever (FMF), 1 Primary myelofibrosis (PM), 1 Polycythemia vena (PA), 1 MDS, and 1 Townes-Brocks syndrome (TBS). The comprehensive clinical results of 8 probands of T8-BD accompanied by genetic variants were summarized in Table 1. The median and IQR 25–75 of age at first visit was 50 (4, 61) years old. The median and IQR 25–75 of age at diagnosis of BD and T8 was 33.5 (7.75, 61) years old and 33.5 (7.75, 57.5) years old, respectively. The median and IQR 25–75 of age at mutation identification was 32 (4.5, 57.5) years old. The BD diagnosis delay was found in 50% patients in this study, and the identification of T8 or genetic mutations tend to occur before BD diagnosis. In addition, the majority of the patients had atypical BD symptoms and failed to fulfill all the BD classification criteria, and thus, were considered to be BD-like disease. The comprehensive information about P1’s family history provided by the participants and/or their family members was presented in Fig. 2A.

**Table 1:** Clinical information of eight T8-BD patients combined with genetic mutations

Patient		P1	P2	P3	P4	P5	P6	P7	P8
Mutation		NRAS G12A	JAK2 V617F	JAK2 V617F	MEFV E148Q	MEFV E148Q	PTPN11 E76A	PTPN11 G503A	SALL1 Nonsense
Nation		China	Japan	Japan	Japan	Japan	Japan	China	England
Gender		Female	Female	Female	Male	Female	Female	Female	Female
Age (years)									
	First visit	0.5	50	61	78	53	4	NA	14
	BD diagnosis	10.5	53	68	78	54	4	5	14
	T8 diagnosis	10.5	53	61	78	54	4	5	14
	Variant identification	1.5	50	61	78	54	4	5	14
BD classification criteria									
	JPN	BD suspected	Incomplete type	BD suspected	Incomplete type	BD suspected	Incomplete type	Incomplete type	BD suspected
	CHN	Incomplete type	Incomplete type	×	×	×	Incomplete type	Incomplete type	Incomplete type
	ICBD	/	√	×	×	×	/	/	/
	PEDBD	×	/	/	/	/	×	×	×
	ISG	×	×	×	×	×	√	×	×
Comorbidity		ALPS	Primary myelofibrosis	Polycythemia vena, trisomy 9	MDS, atypical FMF	MDS, atypical FMF	MDS	None	Townes-Brocks syndrome
Clinical features									
	Oral aphthosis	√	√	NA	NA	√	√	√	√
	Genital ulceration	×	×	×	NA	NA	NA	√	√
	Skin involvement	×	×	NA	√	NA	√	×	NA
	Ocular lesions	×	√	×	NA	NA	×	NA	NA
	Neurological signs	√	NA	NA	NA	NA	×	NA	NA
	Vascular signs	×	NA	√	×	NA	×	NA	×
	Pathergy test	NA	NA	NA	NA	√	√	NA	NA
	Gastrointestinal lesions	Multiple ulcers in ileum, ascending colon, and transverse colon with atypical IBD manifestations	Multiple ulcers in the terminal ileum	Multiple ulcers in the ileocecal region	Erythema, erosions, and mild oedematous mucosa in the caecum and ascending colon	Multiple oval ulcers in the colon, terminal ileum, duodenum, and jejunum	Multiple round ulcerations in the transverse and ascending colon without cobblestone appearance	Intestinal ulcers	NA
	Fever	√	√	×	√	√	√	√	NA
	Arthralgia	√	NA	NA	√	NA	NA	NA	NA
	Hepatosplenomegaly	√	√	NA	NA	NA	NA	√	NA
Laboratory tests									
	Hb (g/dL)	9.00	6.80	10.50	9.70	9.10	11.30	NA	Normal
	WBC (× 10^9^/L)	25.89	15.43	20.56	6.70	1.49	10.02	18.00	Normal
	IgG (mg/dl)	1930.00	NA	NA	NA	NA	Normal	1980.00	NA
	ESR (mm/h)	38.00	NA	NA	NA	NA	NA	54.00	NA
	CRP (mg/dl)	12.95	NA	2.68	8.25	2.16	0.56	4.00	115.00
Medical regimen									
	Hormone	Prednisolone	Prednisolone	Prednisolone	Prednisolone	Prednisolone, hydrocortisone	NA	NA	×
	Immunosuppressants	Mycophenolate mofetil, thalidomide, sirolimus	Ruxolitinib, azacitidine, azathioprine	hydroxyurea	Colchicine	Colchicine, azacitidine	NA	NA	×
	Biologics	Adalimumab	Infliximab	×	Canakinumab	×	NA	NA	×
	Other	×	Antibiotic drugs	Aspirin, cilostazol	×	Antibiotic drugs, γ globulin, benzydamine hydrochloride	NA	NA	Benzydamine hydrochloride, chlorhexidine digluconate
Clinical outcome		Remission	Remission	Remission	Death	Remission	Remission	NA	Remission
Reference		This paper	[72]	[73]	[74]	[62]	[75]	[39]	[76]

BD, Behçet’s Disease; T8, trisomy 8; ALPS, autoimmune lymphoproliferative syndrome; MDS, myelodysplastic syndrome; FMF, Familial Mediterranean fever; IBD, inflammatory bowel disease; Hb, hemoglobin; WBC, white blood cell; CRP, C-reactive protein level; ESR, erythrocyte sedimentation rate

As for the clinical features, the most common manifestations were oral aphthosis (6/6, 100%), intestinal lesions (7/7, 100%), fever (6/7, 85.7%), decreased hemoglobin (Hb) levels (6/7, 85.7%), and increased C-reactive protein (CRP) levels (6/7, 85.7%) in this study. Concerning gastrointestinal lesions, the most frequent presentation was multiple ulcers in terminal ileum and colon, except for the manifestations of erythema, erosions, and mild oedematous mucosa found in P4. The positive rates for genital ulceration, erythema nodosum, uveitis, tinnitus and hearing loss, and vascular involvement were 40%, 40%, 25%, 50%, and 20%, respectively. The vascular sign included portal vein thrombosis (P3) and occasional fibrin plugs combined with neutrophil extravasation occurring in small vessels (P8). Notably, the vascular sign in P8 was identified by biopsy analysis and may not satisfy the clinical diagnostic criteria as thrombosis. The clinical presentations of Oral aphthosis identified in P1 were visualized in Fig. 2B. Pretreatmently, the proportion of “double-negative” T cells (DNT) in peripheral blood was 3.3% of total lymphatic trotting. The elevated levels of white blood cell (WBC), IgG, and erythrocyte sedimentation rate (ESR) were identified, with the percentage of 62.5%, 66.7%, and 100%, respectively. However, decreased WBC concentration was found in P5.

### Genetic characteristics

**Fig. 3:**
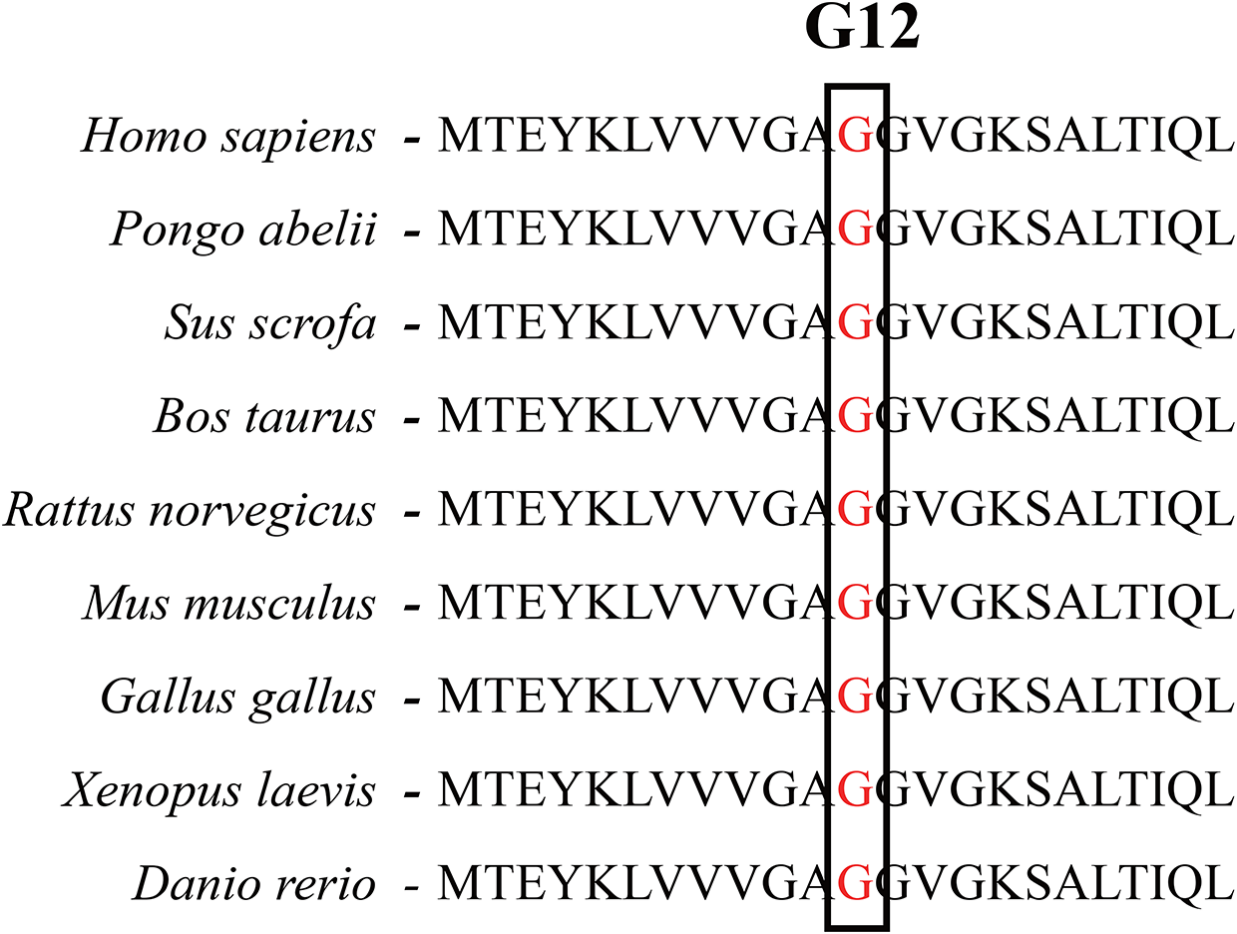
Multiple sequence alignment analysis of NRAS protein. The amino acid residue of Glycine 12 was evolutionarily highly conserved in different species

A total of 6 mutations occurred in *NRAS*, *JAK2*, *MEFV*, *PTPN11*, *SALL1* genes were detected in T8-BD patients in this study, including 5 missense variants and 1 nonsense variant. Notably, two cases harbored identical JAK2 V617F (P2 and P3) mutations and MEFV E148Q (P4 and P5), respectively. Trisomy 9 was also found in P3. As for the reported case (P1) in this study, a known *de novo NRAS* variant was identified by high-throughput sequencing and was further confirmed by Sanger sequencing. In addition, predictive software tools were employed to analyze the potential impacts of amino acid substitution caused by this mutation for pathogenicity assessment, and the ultimate classification of each variant was determined according to the guidelines of American College of Medical Genetics and Genomics (ACMG 2019) [36], resulting in “pathogenic”. According to the results of multiple sequence alignment analysis, the G12A missense mutation affected highly conserved amino acid residue in NRAS, as visualized in Fig. 3. The results of chromosome abnormality and Sanger sequencing were presented in Fig. 2C-D. The detailed pathogenic assessment results of the genetic mutations reported in this study can be found in Table 2. Besides, the prevalence of the NRAS G12A mutation was of low frequency (8.47 × 10^− 7^ in the European population) and was absent in non-European populations, which was first reported in individuals of European ancestry [38]. The detailed prevalence information of the genetic mutations reported in this study was presented in Table 1.

**Table 2:** Genetic mutations identified in in the study and relevant pathogenic assessments

Patient	Gene	Genbank transcript ID	rsID	Variations			CADD score	SIFT	MutationTaster
				Nucleotide change	Amino acid change	Type			
P1	*NRAS*	NM_002524	rs121913237	c.35G > C	p.G12A	Missense	27.7	D	D
P2, P3	*JAK2*	NM_004972	rs77375493	c.1849G > T	p.V617F	Missense	28.7	D	D
P4, P5	*MEFV*	NM_000243	rs3743930	c.442G > C	p.E148Q	Missense	21.0	N	N
P6	*PTPN11*	NM_002834	rs121918465	c.227 A > C	P.E76A	Missense	27.3	D	D
P7	*PTPN11*	NM_002834	rs397507546	c.1508G > C	p.G503A	Missense	32.0	D	D

LD, likely deleterious; D, deleterious; N, neutral

Importantly, the genetic heterogeneity was found in this study. There were variations in clinical manifestations among these 8 patients, even between those two patients carrying the identical mutation (P2 and P3, P4 and P5), implying the complexity of BD pathogenesis and development. To be specific, the age span of patients was extremely distinct, being either children or the elderly in this study. Uveitis and fever were both identified in P2 while P3 denied the existence. The intestinal lesions differed in P4 and P5, and the decreased concentration of WBC was exclusively found in P5, which remain normal in P4. The genotype-phenotype correlations were provided in Table 1.

### Treatment and outcome

In this study, glucocorticoid (GC), immunosuppressants and biologics were administered to 83.3% (5/6), 83.3% (5/6) and 50% (3/6) of cases, respectively. The other drugs include antibiotic drugs (P2), aspirin and cilostazol (P3), antibiotic drugs, γ globulin, and benzydamine hydrochloride (P5), benzydamine hydrochloride and chlorhexidine digluconate (P8). Among them, 2 patients were treated with GC in combination with immunosuppressants, 1 case solely used anti-oral aphthosis medications, and 3 used a combination of GC, immunosuppressants, and biologics. Notably, remission was achieved in 6 patients, except for P4’s death caused by the hemorrhagic shock from diverticular hemorrhage. The detailed information of medication regimens were outlined in Table 1.

## Discussion

Previous studies primarily focused on the autoimmune and autoinflammatory features observed in Trisomy 8-associated Myelodysplastic Syndrome (T8-MDS), while cases of T8 without myelodysplasia have largely been overlooked [24, 39]. Among these, the strong association between T8-MDS and autoinflammatory diseases, especially BD, has been reported [24]. However, no prior cohort studies have simultaneously analyzed cases in which T8, genetic variants, and BD co-occurred. This study was the first to analyze the role of T8 plus genetic mutation in clinical characteristics and disease phenotypes according to the T8-BD patients from The Second Affiliated Hospital and Yuying Children’s Hospital of Wenzhou Medical University and critical literature review. Our study revealed that in patients presenting with BD-like symptoms, combined with T8 and genetic variants, showed unique clinical manifestations to some extent, including higher incidence in children and seniors, in East Asia regions and in women, oral aphthosis, fever and intestinal lesions. Genetic variants in *NRAS*, *JAK2*, *MEFV*, *PTPN11*, *SALL1* gene were identified in T8-BD, and T8 or genetic mutations could be detected before the diagnosis of BD (P1-3). This indicated their potential role in the pathogenesis of BD, and enlightened the early identification of BD, particularly when patients present with atypical symptoms accompanied by inflammatory features, with the assistance of genetic sequencing and karyotyping analysis.

One of the key findings in this study was the identification of a *de novo NRAS* mutation in a pediatric case (P1), which represents a significant contribution to the current understanding of BD-like syndromes associated with T8. NRAS mutations are well-known drivers of oncogenesis and have been associated with hematologic malignancies, including juvenile myelomonocytic leukemia (JMML) and ALPS [37, 40]. This study provided evidence that NRAS mutations, when combined with T8, may also result in an autoinflammatory condition mimicking BD. The patient exhibited hallmark features of BD, including recurrent oral aphthosis and intestinal lesions, but also presented with systemic manifestations typically seen in ALPS, such as cyclic fever, hepatosplenomegaly, and autoimmune cytopenia [41, 42]. This overlap of clinical features may suggest that the *NRAS* mutation, in conjunction with T8, promotes inflammatory pathways that contribute to BD-like symptoms. Despite the early diagnosis of ALPS being made based on the initial presentation of cyclic fever and oral ulcers, the delaying recognition of BD-like symptoms occurred in P1, and the oral ulcers worsened before the accurate treatment was made, which also indicated the diagnosis challenge and early identification in BD.

Notably, mutations in two genes of the RAS/MAPK pathway have been identified in this cohort, namely *NRAS* and *PTPN11* [43]. This indicated the potential role of RAS/MAPK pathway in the pathogenesis and development of BD, which has been previously reported to be one of the underlying pathogenic pathways in BD [21]. NRAS, a member of the RAS family of proteins, functions as a molecular switch that transmits signals from various growth factors and hormones, ultimately influencing cellular responses like proliferation and survival [43, 44]. Mutations in *NRAS* can lead to constitutive activation of this pathway, contributing to inflammatory processes and possibly the manifestation of BD-like symptoms [45]. Similarly, *PTPN11*, which encodes the protein SHP-2, is a key regulator of the RAS/MAPK pathway [46]. Mutations in *PTPN11* are known to lead to hyperactivation of this signaling cascade, promoting cell growth and survival [47].

This study also identified JAK2 V617F and MEFV E148Q mutations in two patients, respectively (P2 and P3; P4 and P5). The MEFV M694V variant, a well-established risk factor for FMF, has been associated with an increased risk of BD [48]. The potential mechanism involves gain-of-function mutations in *MEFV* leading to enhanced immune responsiveness to microbial products, suggesting a role for host-microbe interactions in BD-associated inflammation [49], Polymorphisms of *JAK2* have also been reported to be related to BD [50]. Recently, there have been several studies reporting that Th17 cells—which also secrete IL-17 and IL-22—may be crucial in the BD pathogenesis [51–53]. The JAK2-STAT3 pathway is essential for Th1 and Th17 cell differentiation [54], further implicating its role in immune dysregulation in BD. Moreover, emerging evidence showed that TGF-β activates p38 MAPK to modulate Th17 cell differentiation, further linking JAK2 and MAPK signaling with BD pathogenesis [55, 56].

T8 has been previously associated with hematologic diseases such as MDS and acute myeloid leukemia (AML), and the link bend inflammatory conditions, particularly BD-like symptoms, has gained attention [23]. It has been demonstrated that T8 is an important cause of inflammatory fever, resulting in periodic fever and Behcet’s-like disease [39]. Interestingly, the gastrointestinal lesions seen in these patients, including multiple ulcers in the ileum and colon, is reminiscent of intestinal BD, suggesting that T8 may specifically drive gastrointestinal inflammation [57, 58]. This is particularly significant because intestinal BD is often challenging to diagnose and treat, and the presence of T8 could serve as an important genetic marker in these patients [58, 59].

In terms of the association of phenotypes and genotypes, our study demonstrated the heterogeneity of clinical manifestations among patients with T8-BD, even in those carrying identical mutations (i.e., JAK2 V617F in P2 and P3, MEFV E148Q in P4 and P5). Despite sharing the same JAK2 mutation, P2 exhibited uveitis and fever, whereas P3 did not. Similarly, P4 and P5, both carrying the MEFV mutation, differed in their gastrointestinal manifestations and WBC levels. This variability underscores the challenge in predicting clinical outcomes based solely on genetic findings and suggests that other modifying factors, such as environmental triggers, impaired antioxidant mechanisms, or epigenetic changes, may influence disease severity and progression [3, 18, 60]. Besides, the age distribution of patients with T8-BD also varied significantly, ranging from children to the elderly, which group is rarely seen in BD and differed from the typical onset of BD among 30s and 40s and thus, further complicated the diagnostic process [2]. The racial and regional disparities were also noticed in our cohort compared with typical BD, as the majority in East Asia and in females in this study.

Due to the rarity of T8-BD cases, there is an absence of standard therapy for disease treatment and clinical management. Given the potential role of inflammatory cytokines in T8-BD pathogenesis, dose titration or concomitant biologic agents are often adopted in addition to conventional BD therapy [61, 62]. The treatment outcomes in this cohort highlight the complexity of managing patients with T8-BD. Most patients achieved remission with a combination of GC, immunosuppressants, and biologics such as TNF-α inhibitors (e.g., infliximab, adalimumab). However, the case of P4, who succumbed to hemorrhagic shock due to diverticular hemorrhage, underscores the potential for severe complications in patients with gastrointestinal involvement [63, 64]. This highlights the importance of close monitoring for adverse events, particularly in elder patients with extensive intestinal lesions. The response to biologics indicated that targeting specific inflammatory pathways, such as TNF-α, may be effective in controlling BD-like symptoms in T8-BD, which was consistent with previous studies in BD [65, 66].

The situation might get improved by the development of personalized medicine based on genetic and chromosomal analysis. Patients with underlying genetic mutations (e.g., *NRAS* or *JAK2*) might benefit from combined medication therapy with targeted therapies focusing on the specific molecular pathways involved, such as the RAS/MAPK or JAK-STAT pathways. In fact, p38 MAPK or JAK inhibitors, which are already in clinical use for other inflammatory and hematologic disorders [67–70]. While presenting the new approaches in immunotherapy of BD, Shahneh et al. emphasized that further studies focusing on BD pathogenesis could lead to the development of better therapeutic strategies based on its immunopathogenesis [71]. Additionally, early identification of chromosomal abnormalities could aid in risk stratification and prevent both overtreatment and undertreatment of BD-like symptoms.

The study has some limitations. First, due to the rarity of cases of T8-BD with genetic mutations, there was a bias caused by the limited scale of the cohort. Second, the cohort is predominantly female and of East Asian descent, which may limit the applicability of the findings to other populations. Third, the lack of standardized treatment protocols for T8-BD limits the ability to draw definitive conclusions about therapeutic efficacy. Future multi-center international collaborations and mechanistic studies will be necessary to increase sample size, improve statistical power, and validate these findings across diverse patient groups. In addition, future research should focus on functional studies of the identified genetic variants, the development of targeted therapies, and the standardization of treatment regimens and therapy efficacy assessment.

## Conclusions

In conclusion, this study summarized the clinical features of T8-BD patients combined with genetic mutations and reported a *de novo NRAS* mutation identified in a pediatric BD patients combined with T8. Our findings emphasized the heightened alert on the existence of autoinflammatory disease and the importance of considering both genetic sequencing and chromosomal abnormalities in the diagnostic evaluation of patients with BD-like symptoms, particularly when oral ulcers and systemic inflammation were present, but the patient did not fully meet BD diagnostic criteria. This is helpful for providing more accurate diagnoses, guiding treatment decisions, and improving patient outcomes. Further larger cohort studies are necessary to validate the findings and explore the underlying mechanisms of T8-BD. Future studies can focus on the functional studies of the identified genetic variants, and the development of targeted therapies.

## Electronic Supplementary Material

Below is the link to the electronic supplementary material.

Supplementary Material Table S1

## Data Availability

The original data presented in the study are included in this article. Further inquiries can be directed to the corresponding author.
