# Supplementary material for: Clinical features and novel pathogenic variants of patients with Behçet’s disease like trisomy 8
Source: Orphanet J Rare Dis. 2025 Jul 4;20:340. doi: 10.1186/s13023-025-03878-y (PMC12228353; doi:10.1186/s13023-025-03878-y)
Supplement: Supplementary file 1 — Supplementary Material Table S1 [file 13023_2025_3878_MOESM1_ESM.docx]

**Table S1** Prevalence information of genetic variants identified in patients.

| **Patient** | **Gene** | **Variations** | | **European (non-Finnish)** | | | **East Asian** | | | **South Asian** | | | **African** | | |
| --- | --- | --- | --- | --- | --- | --- | --- | --- | --- | --- | --- | --- | --- | --- | --- |
|  |  |  |  |  |  |  |  |  |  |  |  |  |  |  |  |
|  |  | **Nucleotide change** | **Amino acid change** | **Allele count** | **Allele number** | **Allele frequency** | **Allele count** | **Allele number** | **Allele frequency** | **Allele count** | **Allele number** | **Allele frequency** | **Allele count** | **Allele number** | **Allele frequency** |
| P1 | *NRAS* | c.35G>C | p.G12A | 1 | 1179846 | 8.47×10^-7^ | 0 | 44886 | 0 | 0 | 91082 | 0 | 0 | 75018 | 0 |
| P2, P3 | *JAK2* | c.1849G>T | p.V617F | 356 | 1173540 | 3.03×10^-4^ | 5 | 44774 | 1.11×10^-4^ | 27 | 90798 | 2.97×10^-4^ | 19 | 74656 | 2.54×10^-4^ |
| P4, P5 | *MEFV* | c.442G>C | p.E148Q | 15366 | 1177428 | 1.30×10^-2^ | 11517 | 44726 | 0.25 | 25538 | 90834 | 0.28 | 1230 | 74874 | 1.64×10^-2^ |

The data was retrieved from Genome Aggregation Database (gnomAD, <https://gnomad.broadinstitute.org/)> v4.1.0. The prevalence of PTPN11 E76A and PTPN11 G503A was 0 in gnomAD.
